# Quantifying infants’ everyday restrained experiences in the home using wearable inertial sensors

**DOI:** 10.3758/s13428-026-03185-9

**Published:** 2026-09-30

**Authors:** Hanzhi Wang, Hailey N. Rousey, John M. Franchak

**Affiliations:** Department of Psychology, University of California, Riverside, CA USA

**Keywords:** Infant, Restraint, Everyday experiences, Wearable sensors, Machine learning

## Abstract

Physical restraint—including being held, carried, and restrained in devices—is a common feature of infants’ everyday lives. However, previous survey-based and video-based methods cannot simultaneously provide continuous, full-day accounts of infants’ restrained experiences. This study developed and validated a machine learning model to quantify infants’ restrained time moment-to-moment across the full day in the home environment using wearable sensor data. We used a dataset that includes 146 home-visit sessions from 66 infants, with 30 younger infants aged 4–7 months, and 36 older infants aged 11–14 months. We annotated infants’ restrained states in the first 1.5-h video recording of each session as ground-truth labels. The supervised machine-learning model achieved high accuracy (89%) and substantial kappa agreement (κ = .73) compared with human-coded ground truth. The model showed a slight bias toward overestimating unrestrained periods relative to restrained periods, but this bias was mitigated when we used a longer data-aggregation window. The model also showed convergent validity by corroborating prior studies that showed an age-related decrease in infants’ overall restrained time throughout the day. In short, the current study demonstrated the utility of using wearable sensors to quantify infants’ real-world restrained experiences, offering a new tool for studying how daily restraint influences early development.

## Introduction

In day-to-day life, infants are often held or carried by caregivers (i.e., caregiver restraint), or routinely placed in devices such as high chairs, walkers, or strollers (i.e., device restraint) to ensure safety, facilitate transport, and provide comfort (Birken et al., 2015). Defined as the restriction of an infant’s ability to move or change their posture freely, restraint occurs across a wide range of durations, from brief episodes where a caregiver picks up an infant to reposition them to prolonged periods, such as 30 min in a highchair during mealtime. Ingrained in everyday caregiving routines, restraint fundamentally shapes infants’ experiences by limiting their physical location and altering what they can do, see, hear, or interact with. For example, restraint directly shapes infants’ motor experiences by limiting their muscle movement (e.g., Jiang et al., 2016). Restraint also serves as a learning context that provides different opportunities for object interaction (Franchak et al., 2024a, 2024b), face looking (Kretch, 2025) and speech input (Malachowski et al., 2023; Rousey et al., 2026). Therefore, characterizing infants’ restrained experiences helps profile infants’ daily learning contexts and situate their motor, perceptual, and cognitive development within everyday experiences.

Previous studies have used a variety of methods to document infants’ restrained time in the home. Some used parent surveys, including questionnaires, diaries, or ecological momentary assessment (EMA) (e.g., Abbott & Bartlett, 2001; Franchak, 2019; Karasik et al., 2018). Some used video recording and human annotation (e.g., Lee et al., 2026; Springfield & Lee, 2025; Wang et al., 2025). Although surveys provide daily overviews and video recordings provide continuous time series data, neither method can simultaneously provide full-day, continuous data to account for infants’ restrained experiences. In the current study, we propose that combining wearable sensors and machine learning algorithms can be a solution to record and process long-form, real-time data to characterize infants’ restrained experiences.

### Previous measurements of restraint

Despite different approaches, prior work converges to show that restraint is common in infant daily life, and that the time infants spend restrained changes over development. In terms of caregiver restraint, 2- to 6-month-old infants spent around 40% of their awake time being held or carried (Malachowski et al., 2023; Siddicky et al., 2020). This percentage reduced to around 19% for older 10- to 13-month-olds (Franchak et al., 2024a, 2024b). Regarding device restraint, younger 4- to 6-month-olds spent about 23% of awake time in seating devices (Malachowski et al., 2023). Meanwhile, the total amount of device restraint time ranged widely from 0 to 16 h per day for infants younger than 5 months (Callahan & Sisler, 1997), but decreased with age from 2 to 6 months (Carson et al., 2022). Older 10- to 13-month-olds spent around 27% of awake time in restraining furniture and devices (Franchak et al., 2024a, 2024b).

Many other studies have documented some portion of infant restrained time, but they differed in restraint definitions, making it difficult to compare results across studies. Some definitions only focused on caregiver holding and carrying (e.g., Airaksinen et al., 2024; Franchak et al., 2021, 2024a, 2024b; Yao et al., 2019), some only focused on seating devices (e.g., Callahan & Sisler, 1997), some looked at different surfaces of placement (e.g., lap, cradle, and seat) (e.g., Graciosa et al., 2024), some focused broadly on containment equipment (e.g., Bartlett & Kneale Fanning, 2003; Carson et al., 2022; Hesketh et al., 2015; Karasik et al., 2022), and others covered a wider range of restraint like furniture placement, seating devices and holding (Franchak et al., 2024a, 2024b; Malachowski et al., 2023; Siddicky et al., 2021; Springfield & Lee, 2025).

In the current study, we take a broader scope and consider any practices that limit infants’ freedom of movement as restraint. We do not predefine a list of restraint practices but focus on infants’ real-time opportunities for movement. By including both caregiver and device restraint, we can understand how restraint as a whole constrains and facilitates infants’ real-time opportunities for action, perception, and interaction.

### Restraint can constrain and facilitate experiences

As an external constraint, restraint can be detrimental. In the domain of motor development, various studies have shown that higher levels of both device and caregiver restraint experienced by infants as early as 8 months are correlated with decreased proficiency and temporary delays in the acquisition of motor skills (e.g., sitting, crawling, and walking) in both full-term and low-risk preterm infants (Abbott & Bartlett, 2001; Bartlett & Kneale Fanning, 2003; Carson et al., 2022; Karasik et al., 2023; Pin et al., 2007). Kinesiology measurements suggest that reduced body movement might be the underlying mechanism. For example, being tucked in a car seat significantly reduces infants’ spinal muscle activity (Siddicky et al., 2021), hip position and muscle activity (Siddicky et al., 2020), and leg movement quantity (Jiang et al., 2016) compared to being unrestrained. Since motor skill acquisition has cascading effects on infants’ object learning (Soska et al., 2010), spatial cognition (Clearfield, 2004; Frick & Möhring, 2013), and language development (e.g., Oudgenoeg-Paz et al., 2015; Walle & Campos, 2014), excessive restraint time could have downstream consequences if restraint delays motor development. Restraint is also linked to limitations in real-time experiences in other developmental domains. For example, infants have fewer opportunities for object interactions when restrained compared to unrestrained (Franchak et al., 2024a, 2024b). Infants are also exposed to a reduced quantity and consistency of adult language when restrained in devices (Malachowski et al., 2023).

However, some aspects of restraint can be positive. For infants with limited motor control, restraint can facilitate learning by stabilizing their body posture. For example, pre-sitting infants often rely on their hands to maintain posture, which can limit opportunities for visual-manual coordinated exploration. Placing infants in supported sitting positions, such as on a caregiver’s lap or in a highchair, can free their hands and support higher-quality object exploration compared to unsupported sitting (Kretch, 2025; Woods & Wilcox, 2013) or compared to non-sitting positions (Soska & Adolph, 2014). Having a free hand to point at objects also elicits more verbal responses from caregivers (Wu & Gros-Louis, 2015). Moreover, restraint can increase certain learning inputs. Being held and carried by caregivers is associated with more speech input (Rousey et al., 2026). Some restraint devices (e.g., high chairs) and caregiver holding can place infants high off the ground, providing them with richer visual input of faces, distant toys, and elevated portions of their environment compared to when prone on the ground (Franchak et al., 2018; Kretch et al., 2014; Luo & Franchak, 2020).

Therefore, restraint cannot be categorized as either harmful or beneficial. Rather, these examples underscore that real-time observation of restraint is essential for understanding how restraint shapes infants’ multimodal learning opportunities from moment to moment.

### Limitations of previous research methods

Researchers have used survey methods, including traditional retrospective reports and ecological momentary assessment (EMA), to quantify infants’ restrained time, but survey methods have limitations. Retrospective parent report asks parents to estimate their infant’s average daily restrained time over the past few weeks or months (Abbott & Bartlett, 2001; Bartlett & Kneale Fanning, 2003; Callahan & Sisler, 1997; Carson et al., 2022; Hesketh et al., 2015; Karasik et al., 2018; Siddicky et al., 2020). Despite its widespread use, retrospective reports lack precision because they rely on parents’ memory; it is difficult to recall and sum every bout of restraint and then estimate overall time (Bradburn et al., 1987). There are exceptions where caregivers were asked to complete structured daily diaries reflecting on the previous day’s restraint practices in short intervals (e.g., every five minutes) (Karasik et al., 2018; Majnemer & Barr, 2005). However, this method might still be sensitive to memory loss and thus not as accurate in providing data. EMA survey techniques reduce caregivers’ memory burden by sending momentary phone-survey prompts to caregivers multiple times (e.g., 5, 10, or 12 times) randomly throughout a day (Franchak, 2019; Franchak et al., 2024a, 2024b; Malachowski et al., 2023). Despite the benefits of gathering immediate observations, EMA cannot provide continuous time-series data. Thus, a method is needed that provides time-sequence information without relying on parent report.

Video recording and human annotation overcome the above drawbacks of survey-based methods, but video is subject to other limitations. Video recording by handheld cameras can be obtrusive and potentially impact caregivers’ and infants’ behaviors. Stationary cameras (e.g., fixed on a tripod) may lose sight of the infant due to restricted fields of view or obstructions from furniture and people in the room. Furthermore, the video annotation process can be labor-intensive and time-consuming, which limits its use in large-scale or long-duration studies. Indeed, prior work that videotaped and manually annotated infants’ restraint had short recording durations (e.g., half an hour in Lee et al. (2026) and Springfield and Lee (2025), and one h in Wang et al. (2025)). Additionally, within the short duration of recording, researchers tend to record the period when infants are awake and active. In contrast, periods when the infants are restrained, like seated in front of the TV or seated in high chairs, would likely be skipped. Thus, video recording is subject to sampling bias and might not be representative of infants’ whole-day experiences.

### Promises and challenges of wearable sensors

A system that uses wearable sensors (i.e., inertial movement units) to record real-time movement data and machine learning models to classify movement categories can overcome the limitations listed above. Wearable sensors capture behavior-agnostic raw movement data, including linear acceleration from accelerometers and angular velocity from gyroscopes. Researchers define target behaviors and provide ground-truth labels based on video footage. A supervised machine learning model is then trained to map the movement data onto predefined behavioral categories.

This system addresses the aforementioned limitations of survey and video methods in the following ways. First, by collecting infants’ movement data through sensors, the system can objectively capture infants’ motion without relying on a respondent’s report. Second, the sensors can provide moment-to-moment observation that is continuous and rich in time-sequence information. Third, sensors embedded in baby garments are mobile and unobtrusive—an experimenter does not need to follow the infant with a camera to record movements. Fourth, the system can record and process full-day and even multi-day data because the sensors have sufficient battery life and machine learning models can classify data automatically.

Over the past decade, wearable sensors have increasingly been used to record and classify movement categories to gain these advantages. The application of wearable sensors progressed from adults (Arif & Kattan, 2015; Preece et al., 2009), to children (Nam & Park, 2013; Ren et al., 2016; Stewart et al., 2018), and then to infants (Airaksinen et al., 2020, 2024; Franchak et al., 2021, 2024a, 2024b; Yao et al., 2019). For infants, there are established models that classify infants’ body postures—categories include supine, prone, sitting, and being held (Airaksinen et al., 2020; Franchak et al., 2021, 2024a, 2024b; Kretch et al., 2026). There are also models that detect only holding and carrying periods (Airaksinen et al., 2024; Yao et al., 2019).

However, no established model detects infants’ general restrained periods (both device-restrained and caregiver-restrained) from wearable sensor data. Detecting restrained periods might be more challenging than classifying body postures. First, infants may show similar body movements whether restrained or not, making restrained and unrestrained classes more difficult to differentiate. For example, infants might kick their legs when they are lying unrestrained and also when they are strapped in a highchair. Possible distinctions might be manifested in a reduced range of motion or repetitive motion due to constraints, which require high-precision sensors and sensitive models to detect. Second, there are lots of possible restraint forms infants can present—a caregiver can hold an infant in an upright position or lay the infant in arms while breastfeeding; seating restraint can happen in countless infant seats, which vary in affordances of moving (Alghamdi et al., 2024). Furthermore, naturalistic home environments often impair model performance (Airaksinen et al., 2024; Franchak et al., 2024a, 2024b) compared to controlled laboratory settings (Airaksinen et al., 2020; Franchak et al., 2021), likely because infants’ movements at home are more variable. Accordingly, the wearable sensor system should also be robust enough to generalize across heterogeneous restraint scenarios.

One way to meet the dual requirement of sensitivity and robustness is to adjust the temporal scale of predictions. Wearable sensor studies usually use a windowing process (Airaksinen et al., 2020, 2024; Franchak et al., 2021, 2024a, 2024b; Yao et al., 2019), where they apply a sliding window of a certain length to the raw movement data and summarize motion features, such as mean, standard deviation, and correlation, within each window. The machine-learning model then uses the motion features to predict the behavioral category at the window center. Theoretically, shorter windows offer higher temporal resolution and are more sensitive to abrupt changes, but fewer samples per window may leave the model without enough context to classify accurately. In contrast, longer windows can incorporate more data and provide more robust prediction, but may lack temporal sensitivity or may introduce irrelevant data from heterogeneous movement within an event. Studies with different prediction purposes used different window lengths. For example, window lengths of 2.3–4 s were validated for classifying infants’ body positions (Airaksinen et al., 2020; Franchak et al., 2021, 2024a, 2024b), and window lengths of 1.15–10 s were validated for detecting holding and carrying episodes (Airaksinen et al., 2024; Yao et al., 2019). Given the lack of evidence on optimal window lengths for detecting general restrained episodes, this study evaluates how window length affects model performance on restraint classification.

### Current study

We propose that wearable sensors offer a more objective, unobtrusive, information-rich, and efficient approach to capturing infants’ full-day restrained experiences in natural settings compared to prior methods. To test the approach, we used an existing dataset with infants aged from 4 to 14 months (Franchak, 2023). The dataset consists of 146 sessions of full-day wearable sensor recording and 1.5-h video recording of infants’ movement in the home. We coded infants’ restrained status from the videos to provide ground truth labels. We then trained a supervised machine learning model based on the human-annotated labels.

We validated the model in two ways. First, we compared how accurately model predictions matched human annotation for the 1.5 h of each video-recorded session. We also examined how varying window length influences model performance. Second, we applied the model to full-day data to estimate the proportion of time each infant spent restrained. We examined the convergent validity of the model against past research by testing whether restrained time decreased with age (Carson et al., 2022; Franchak et al., 2024a, 2024b).

## Method

### Dataset

The dataset is shared on a web-based repository (Franchak, 2023; https://databrary.org/volume/1637) hosted by Databrary. The dataset consists of two cohorts of infants: 30 younger infants aged 4 to 7 months, and 36 older infants aged 11 to 14 months. Among the 66 infants, 39% (*n* = 26) participated only once, while 61% (*n* = 40) participated 2–4 times, resulting in a total of 146 sessions. Thirty-five of the infants are female and thirty-one are male. Caregivers reported the infants’ race as White (*n* = 23), Hispanic or Latinx (*n* = 8), Asian (*n* = 3), African American (*n* = 2), more than one race (*n* = 15), or not fitting into the above races or chose not to answer (*n* = 15). More detailed descriptions of the sample are in other publications derived from the dataset (Franchak et al., 2026; Rousey et al., 2026).

### Apparatus

In the dataset, a special infant garment was customized to secure four lightweight wearable sensors (MC10 Biostamp) at four specific locations (i.e., right thigh, left thigh, right ankle, and left ankle). Each sensor collects raw data as accelerometer signals (in *x-*, *y-*, and *z*-orientations) and gyroscope signals (in roll, pitch, and yaw orientations). The sampling rate of 62.5-Hz allows for high-resolution motion recording, and the battery life of more than 1 day and sufficient built-in storage allow for daylong recording. An action camera fixed on a mini tripod (GoPro HERO9 Black) was used to record synchronized third-person video of the first 1.5 h into the session as ground truth.

### Procedure

On the day of the home visit, an experimenter turned on the equipment by the doorstep of the participant’s house and synchronized the video camera and wearable sensors by dropping the sensors in front of the camera. This action left a distinct time stamp in both the video footage and the sensors’ data. The experimenter then left the house and instructed the caregiver over the phone to set up the camera in the room where the infant spent the most time in, and to move it as needed to ensure the infant remained in view. The caregiver was instructed to put the garment on the infant and complete a brief set of structured movement tasks for model-training purposes. Caregivers were asked to position the infant supine, prone, sitting, and upright; to hold the infant while walking; and to place the infant in a commonly used restraint device, such as a highchair. Each position lasted approximately 1–4 min, depending on the activity and the infant’s tolerance. After this, the caregiver was asked to play with the infant on the floor for 10 min before they continued their daily routines. The caregiver was also provided with a paper log form to manually record the exact clock time (both start and end times) when the infant was napping or when the garment was removed for reasons such as diaper changes, baths, or going out running errands. These times were later excluded from estimates of daily restrained time. For convenience, we refer to the remaining, included recording time as infants’ “awake time.”

### Human annotation of restraint

Using the 1.5-h videos, our team annotated the periods when the infant was restrained or unrestrained using Datavyu software (https://datavyu.org/). Restrained and unrestrained were defined as a binary category, meaning that all times were coded as one of the two categories. A period was considered missing if the infant went out of view; missing periods were excluded from model training and evaluation. Restraint was defined as when infants could not initiate or control their own movements, which included being held or carried by caregivers, or being restrained in devices. Unrestrained periods, in contrast, were when infants could freely change their body position or locomote. For example, an infant was considered restrained when strapped into a highchair, but not when they were placed in a big crib and free to change position. Each video file was coded by two coders: a primary coder annotated the whole 1.5-h video, and an independent reliability coder annotated the first 30 min of each video. Inter-rater reliability was calculated as the proportion of video frames where the two coders coded the same label. The human annotation reached a high agreement with an overall accuracy of 98.79%, ranging from 89.28% to 99.99%, and an average kappa of 0.98, ranging from 0.80 to 1.00.

### Model classification of restraint

The supervised machine-learning classification process followed procedures similar to prior work (Franchak et al., 2021, 2024a, 2024b; Nam & Park, 2013). First, we aligned the time series of human annotations and sensor data by matching the synchronization timestamp in the video and sensors. This resulted in a dataset with human-coded labels and motion data from four sensors—each capturing acceleration (*x*, *y*, *z*) and angular velocity (roll, pitch, yaw)—available at every sampling point. We then aggregated raw motion data within the sliding window of *n* seconds into motion features, which included statistical features—mean, median, minimum, 25th percentile, 75th percentile, maximum, skewness, kurtosis, standard deviation, and sum—for all six signals across the four sensors, resulting in 240 features. We added 196 additional features based on cross-sensor and cross-orientation metrics such as correlations and pairwise differences, resulting in 436 features in total within each window. We varied the window length *n* from 4 to 16 s and 30 s.

We started the windowing process from the synchronization point and slid the window every *n*/2 s. Consequently, each time point spaced every *n*/2 s contains 436 motion features, aggregated from the *n*/2 s before and after that point. For model training and testing, we included only windows where the infant remained in a single status for over 90% of the time and labeled the window with that status. For the 4-s window length, 1.78% of windows were excluded, leaving 304,542 windows. For the 16-s window length, 5.75% of windows were excluded, leaving 73,002 windows. For the 30-s window length, 8.99% of windows were excluded, leaving 37,563 windows.

The third step was to train and validate supervised machine learning models that classify the motion features into restrained status. We applied the random forest algorithm (Breiman, 2001) in modeling and used a leave-one-out cross-validation approach, where we held out one session at each iteration as a testing set and trained the model using the other sessions. To evaluate how well the model generalized to unseen data compared to the human annotation, we calculated performance metrics at each iteration, including accuracy, kappa, correlation between actual and predicted prevalence, sensitivity, positive predictive value (PPV), and binary F1. Accuracy shows the proportion of windows for which the model prediction agreed with the human annotation. Kappa adjusts the accuracy for chance-level agreement. Prevalence correlation examines whether the proportion of windows classified as restrained (i.e., prevalence of restraint) predicted by the model correlates with the one annotated by humans. Sensitivity reflects the proportion of true restrained observations (marked by humans) correctly detected by the model, whereas PPV reflects the proportion of predicted restrained observations that were truly restrained. Binary F1 summarizes the balance between sensitivity and PPV. For all metrics, higher values indicate better model performance. Based on prior work and benchmarks in the field (Airaksinen et al., 2020, 2024; Franchak et al., 2021, 2024a, 2024b; Landis & Koch, 1997; Yao et al., 2019), we interpreted accuracy above 85% and kappa values above 0.70 as high agreement. We categorized a prevalence correlation coefficient above 0.80 as a strong correlation, and values above 0.80 for sensitivity, PPV, and F1 as high performance.

Whereas accuracy, kappa, and prevalence correlation reflect overall model performance, sensitivity, PPV, and F1 are category-specific metrics. Especially for sensitivity and PPV, the differences in the values between the restrained and unrestrained categories can reveal potential prediction bias. If sensitivity is higher for the restrained than the unrestrained category, the model is better at detecting true restrained periods than unrestrained periods. If the PPV is higher for the restrained than the unrestrained category, the model-predicted restrained periods are more likely to be credible than the predicted unrestrained periods. If a model shows higher sensitivity and lower PPV for the restrained category, the model is biased toward predicting restraint by capturing the true restrained periods but also producing false alarms. We therefore compared sensitivity and PPV across categories as indicators of prediction bias.

The leave-one-out cross-validation could inform us which window length brought the optimal result. The overall accuracy and kappa value were the primary criteria, with minimizing prediction bias serving as a secondary criterion in the case that different window lengths had similar overall metrics. After the window length was picked, we trained a final model using all the human annotations to process the whole-day sensor data of all sessions. We examined the model’s convergent validity by replicating a previously reported effect that restraint time decreases with age (Carson et al., 2022; Franchak et al., 2024a, 2024b).

### Reproducibility and data sharing

The machine-learning process was conducted in Julia version 1.11.1 using the DecisionTree package (Sadeghi et al., 2022). This reproducible manuscript, including the model evaluation, figures, and statistical analyses, was made with Quarto version 1.5.57 (Posit, PBC, 2024) using R version 4.5.0 (R Core Team, 2025) with the R packages tidyverse (Wickham, 2023), flextable (Gohel & Skintzos, 2025), and papaja (Aust & Barth, 2024), as well as the APA Quarto template (Schneider, n.d.). Human annotation results, processing scripts, and analysis code are publicly shared on OSF (https://osf.io/a698g/overview?view_only=cc1483debc864757a99a059bb4b055d1).

## Results

**Fig. 1:**
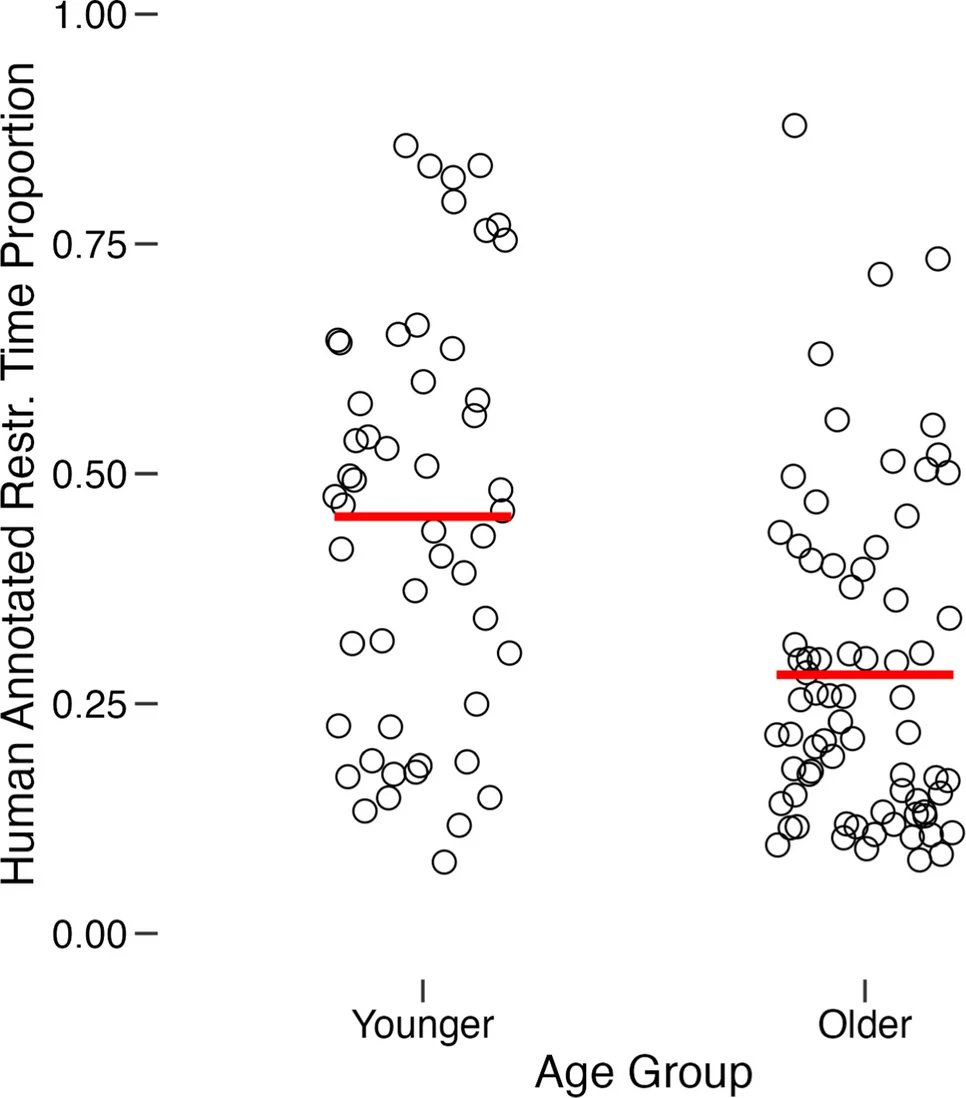
Human-annotated restrained proportion from the 1.5-h video recording. *Note*. Each *circle* denotes one session. The *horizontal line* denotes the mean of restrained time proportion among the 1.5 h for each session

The first part of the validation was based on the first 1.5 h of each of the 144 sessions for which human annotations were available. We excluded two sessions because no video was available to provide ground truth. As shown in Fig. 1, infants were annotated as restrained for an average of 35.29% (*SD* = 21.16%) and unrestrained for an average of 47.52% (*SD* = 21.92%) time among the 1.5-h recording period (the rest of the time was coded as missing), with substantial individual variability. We report the model performance using accuracy, kappa, sensitivity, PPV, binary F1, and prevalence of restraint as compared to human annotation. We also report the model’s prediction bias by comparing the sensitivity and PPV between the two categories.

**Fig. 2:**
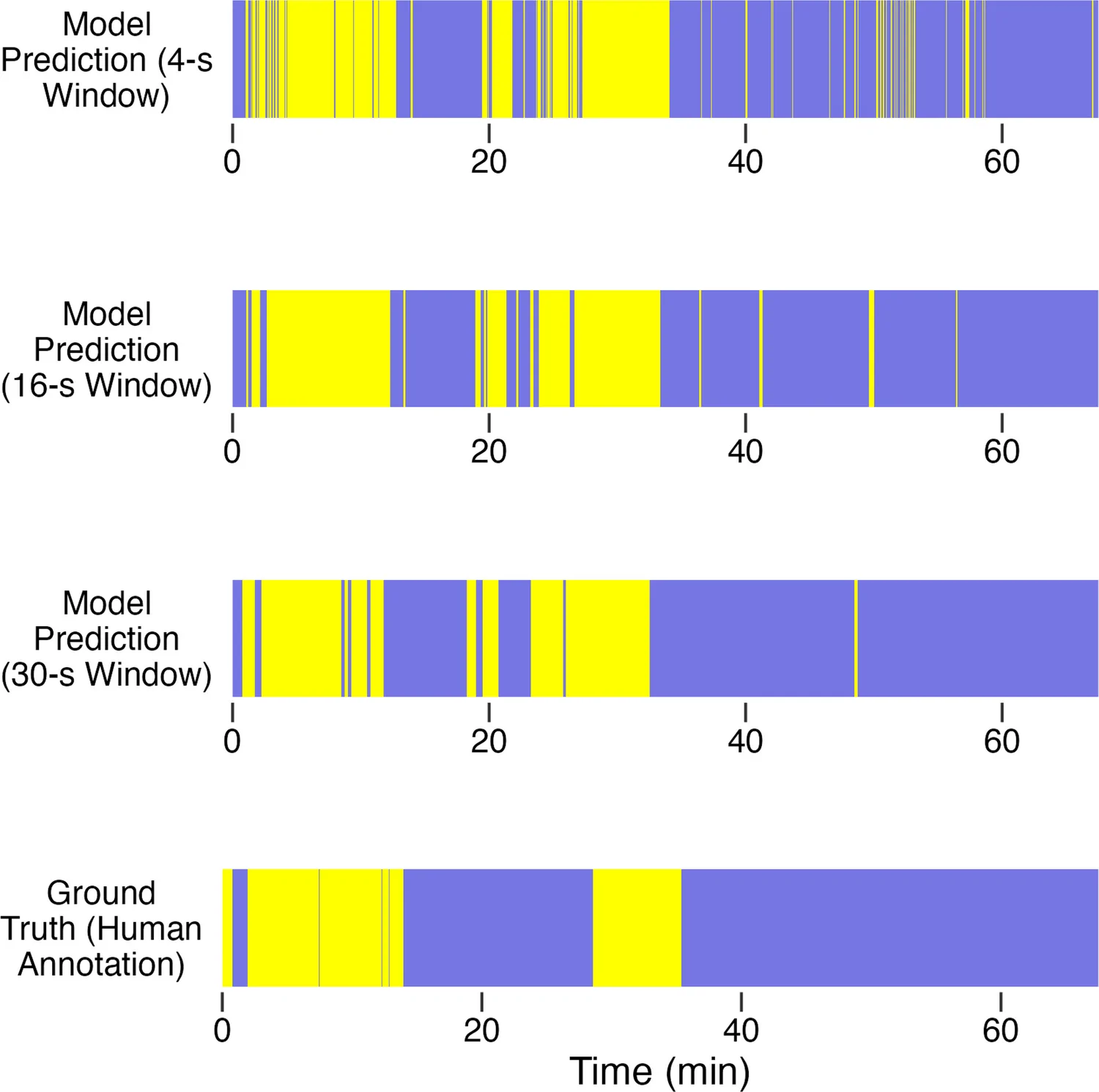
Exemplar timeline of model prediction and human annotation. *Note*. This session was chosen as an example because it represents the average model accuracy. *Purple bars* in each timeline represent model predictions/human annotation of “restrained” and *yellow bars* represent “unrestrained”

The second part of the validation was based on the full-day sensor recordings. We report whether the predictions made by the model with optimal window length converge with past results (Carson et al., 2022; Franchak et al., 2024a, 2024b) to show that older infants had less restrained time than younger infants.

### Model prediction compared to human annotation

Figure 2 shows an exemplar timeline of restraint from one session, comparing human ground-truth annotation with model predictions across the three window lengths. Overall, the model showed strong agreement with human annotation. To quantify this agreement, we calculated the model performance metrics, which were then averaged across sessions.

**Fig. 3:**
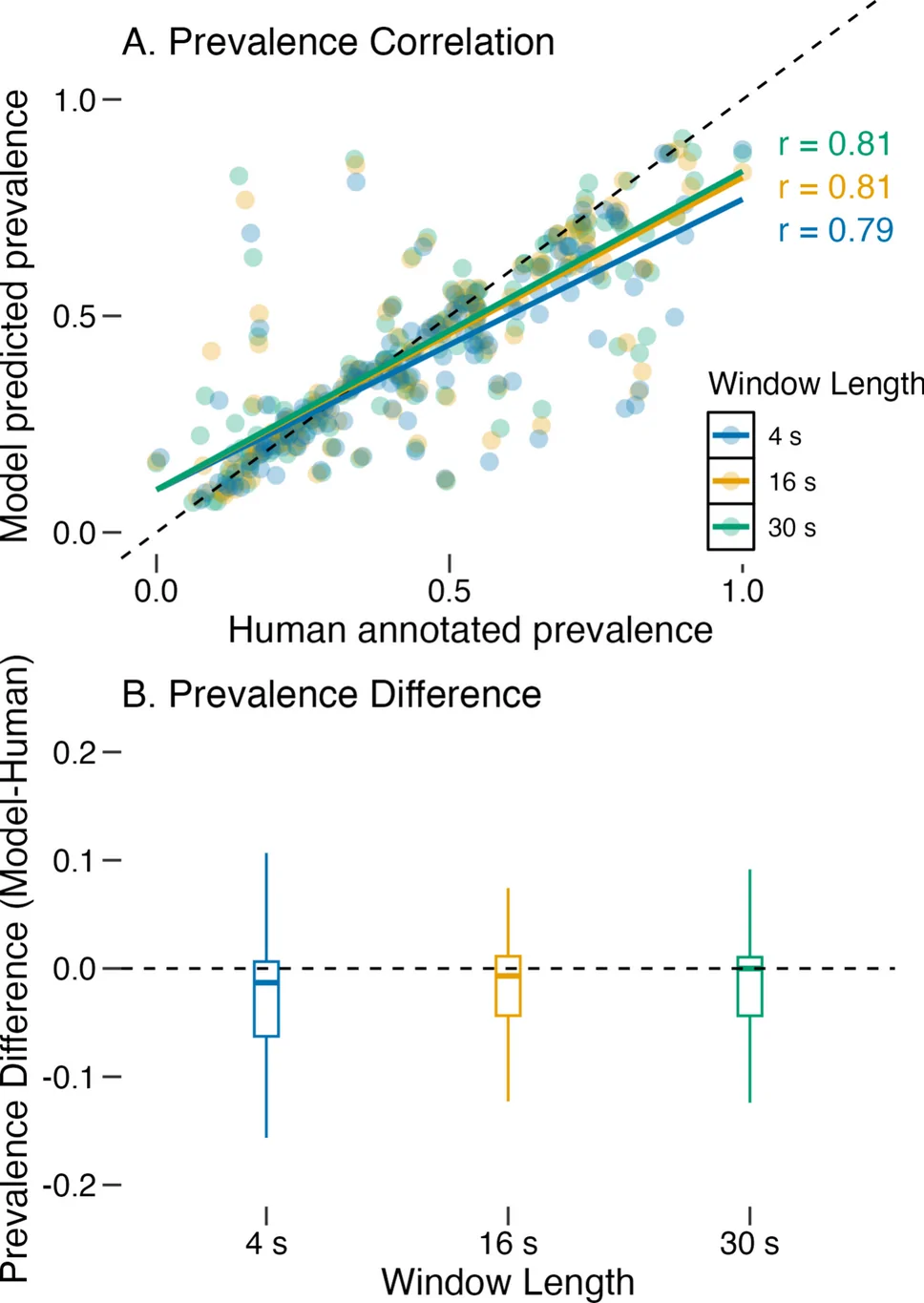
Agreement between model-predicted and human-annotated prevalence of restraint. *Note*. Prevalence is defined as the proportion of windows predicted (or annotated) as restrained among all windows. **A** shows the correlation of prevalence between model prediction and human annotation. The *black dashed identity line* indicates perfect agreement. Each *circle* represents one session for a specific window length (*blue* = 4 s, *yellow* = 16 s, and *green* = 30 s). **B** shows the difference of prevalence between human annotation and model prediction. A negative prevalence difference indicates the model estimated less amount of time infants were restrained compared to human annotation. The *horizontal line in the box* denotes the median, the *borderlines of the box* denote the first (Q1) quartile and the third (Q3) quartile, and the *lines extended* denote the min and max data points within the range of 1.5 IQR (i.e., Q3–Q1)

#### Overall model performance

Table 1 shows that all window lengths reached high accuracy (0.87 for 4 s, 0.88 for 16 s, and 0.89 for 30 s), and high kappa values (0.70 for 4 s, 0.72 for 16 s, and 0.73 for 30 s). For both categories, all window lengths presented high sensitivity (0.82–0.86 for restrained, and 0.91–0.92 for unrestrained), high PPV (0.88–0.88 for restrained, and 0.83–0.86 for unrestrained), and high F1 scores (0.83–0.86 for restrained, and 0.86–0.87 for unrestrained). Moreover, the three window lengths did not significantly differ for any of the metrics (*p*s ≥.057).

**Table 1:** Performance metrics according to window length (4 s, 16 s, and 30 s)

Metric	Category	4 s	16 s	30 s	*F*	*df*	*p*
Accuracy		0.87	0.88	0.89	0.58	(2, 423)	.561
Kappa		0.70	0.72	0.73	0.44	(2, 423)	.644
Sensitivity	Restrained	0.81	0.85	0.86	2.88	(2, 421)	.057
	Unrestrained	0.92	0.91	0.91	0.32	(2, 420)	.727
PPV	Restrained	0.88	0.88	0.88	0.02	(2, 423)	.982
	Unrestrained	0.83	0.85	0.86	0.68	(2, 423)	.505
F1	Restrained	0.83	0.85	0.86	1.14	(2, 421)	.322
	Unrestrained	0.86	0.87	0.87	0.12	(2, 420)	.890

*F*, *df*, and *p* value indicate one-way ANOVA on each metric comparing across the three window lengths.

At the session level, all three window lengths also provided reliable estimates of the overall restrained time as compared to human annotation (Fig. 3A), as the prevalence predicted by the model was highly correlated with the prevalence annotated by human coders (*r* = 0.79 for 4 s, *r* = 0.81 for 16 s, and *r* = 0.81 for 30 s). Thus, model predictions are capable of capturing individual differences in overall restrained time across infants.

**Fig. 4:**
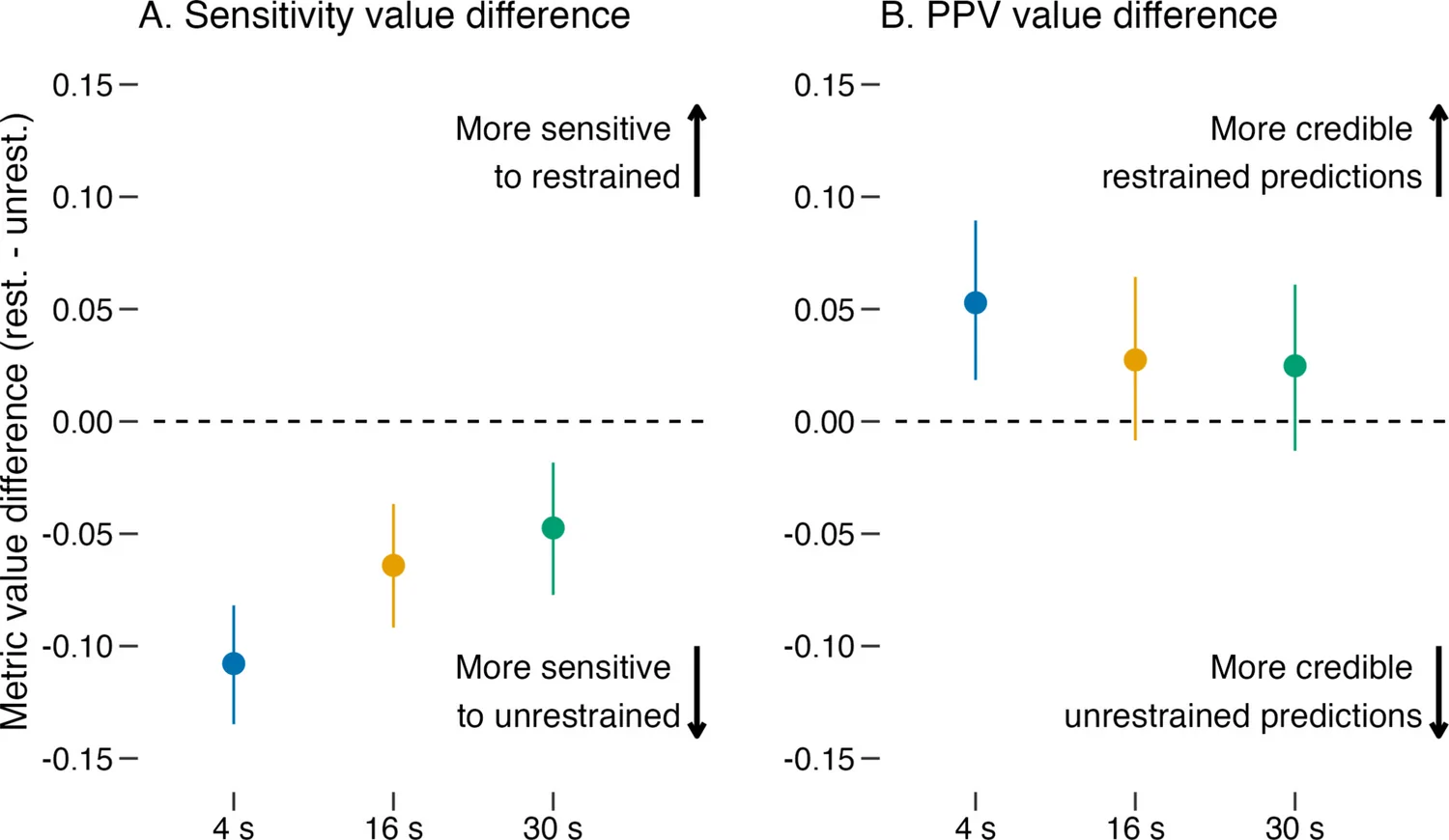
Differences in sensitivity and PPV between restrained and unrestrained prediction according to window length. *Note*. Each *vertical line* indicates the 95% CI of the metric’s difference between restrained prediction and unrestrained prediction. The *dot* on each line denotes the mean of the difference

#### Model bias toward predicting unrestrained periods

Despite the overall strong accuracy, the model was biased towards predicting more unrestrained periods than restrained periods, regardless of the window length. Figure 3 B shows that even though the model predicted prevalence highly correlated with the ground truth, the model tended to underestimate the occurrences of restrained periods. To quantify this bias, we calculated the prevalence difference by subtracting human-annotated restraint prevalence from model-predicted restraint prevalence. Thus, negative values indicate underestimation by the model. The prevalence difference was – 4.70% for the 4-s window, 95% CI [– 6.44%, – 2.97%]; – 2.40% for the 16-s window, 95% CI [– 4.07%, – 0.65%]; and – 1.70% for the 30-s window, 95% CI [– 3.46%, 0.11%]. Although the prevalence difference was not significantly different between the three window lengths (*F*(2, 423) = 1.82, *p* =.16), the 30-s window presented a slightly smaller prevalence difference.

Category-specific metrics further demonstrated this bias. Figure 4 shows the differences in sensitivity and PPV between the restrained and unrestrained categories. The model was less sensitive in detecting true restrained periods than in detecting true unrestrained periods, as shown by the negative value when subtracting the sensitivity of unrestrained prediction from restrained prediction (Fig. 4A). The sensitivity difference was – 0.11 for the 4-s window, 95% CI [– 0.13, – 0.08]; – 0.06 for the 16-s window, 95% CI [– 0.09, – 0.04]; and – 0.05 for the 30-s window, 95% CI [– 0.08, – 0.02]. The sensitivity difference was not significantly different between the three window lengths (*F*(2, 418) = 2.80, *p* =.06). Meanwhile, the model predicted restrained periods were more credible than the predicted unrestrained periods, as shown by the positive value when subtracting the PPV of unrestrained prediction from restrained prediction (Fig. 4B). The PPV difference was 0.05 for the 4-s window, 95% CI [0.02, 0.09]; 0.03 for the 16-s window, 95% CI [– 0.01, 0.06]; and 0.02 for the 30-s window, 95% CI [– 0.01, 0.06]. The PPV difference was not significantly different between the three window lengths (*F*(2, 418) = 0.26, *p* =.77).

**Fig. 5:**
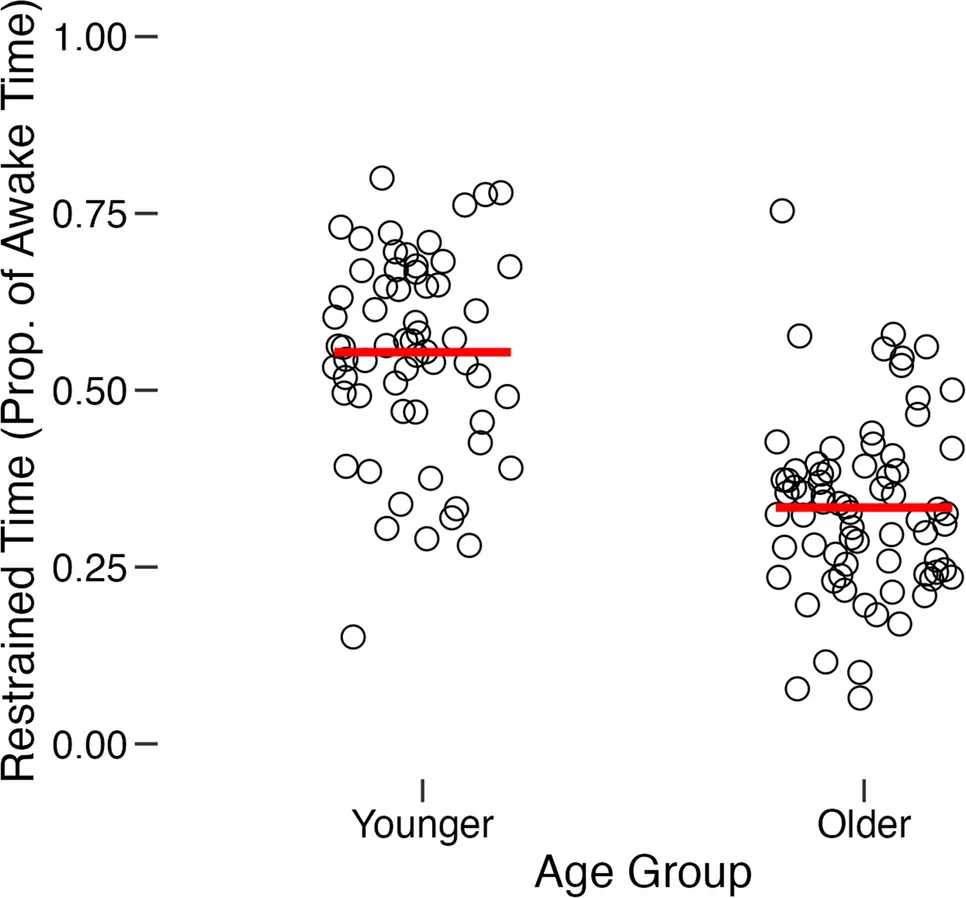
Younger versus older infants’ full-day restrained time. *Note*. Each *circle* denotes one session. The *horizontal red line* denotes the group mean of restrained time proportion (out of awake time)

However, although differences among window lengths were not statistically significant, the 30-s window showed the most favorable descriptive pattern, with the smallest prevalence difference of restraint compared with human annotation and the smallest category asymmetry in sensitivity and PPV. We therefore selected the 30-s window as the optimal window length for full-day prediction.

### Model estimates replicate previous studies

We applied the model with the 30-s window length to the full-day recording for all sessions. We excluded 11 sessions whose recording time was less than 3 h, which were useful for training and validating the models but insufficient for measuring day-long experiences. We also excluded another two sessions where the family took the garment off for a substantial portion of time but did not log the time stamp of removal. In the remaining 133 sessions, infants had an average awake time of 6.18 h (*SD* = 1.88).

Model-estimated restrained time converged with previous studies (Carson et al., 2022; Franchak et al., 2024a, 2024b). Younger infants aged from 4 to 7 months spent *M* = 55.38% (*SD* = 14.03%) of awake time restrained, and older infants aged from 11 to 14 months spent *M* = 33.42% (*SD* = 12.49%) of awake time restrained. Moreover, Fig. 5 shows a significant group difference in restrained time between older infants and younger infants (*b* = – 0.21, *SE* = 0.03, *p* <.001), supporting the convergent validity of the current system.

## Discussion

In the current study, we developed and validated a machine learning algorithm to detect infants’ restrained experiences from wearable sensors in the home environment. The model presented high accuracy and substantial kappa agreement compared to human-annotated ground truth, which are comparable to other wearable sensor algorithms classifying other infant movement categories in the home (Airaksinen et al., 2020, 2024; Franchak et al., 2024a, 2024b; Yao et al., 2019). The model presented a slight bias towards unrestrained—the model tended to overestimate unrestrained periods, but was not as credible when labeling a period as unrestrained. The bias might be due to the inherent imbalance in the training set where unrestrained periods were more prevalent than restrained periods, indicated by human-annotated prevalence (Fig. 2). Using a longer window length (i.e., 30 s, rather than 16 s or 4 s) mitigated but did not eliminate the prediction bias. Ultimately, the model with 30-s window length presented convergent validity in the overall estimate of restrained time and age-related decrease aligned with previous findings (Carson et al., 2022; Franchak et al., 2024a, 2024b).

Why could a longer window length (i.e., 30 s) provide more balanced detection? One explanation is that a longer window length can better capture the contextual information of restraint. Consider the contextual nature of restraint: infants may exhibit similar body movement whether restrained or not for most of the time, but one subtle occurrence of distinctive movement can signal restraint. For example, an infant can be sitting on the floor unrestrained, on a caregiver’s lap restrained, or strapped in a seat restrained. It is hard to distinguish if the infant is restrained while sitting still, but when the infant moves their limbs, restraint may present differences, such as a stronger damping in the motion decay because the support (i.e., caregiver’s laps, or seats) absorbs the motion. These differences only present themselves in the episodes of movement, not throughout the whole period. Thus, a longer window length has a better chance of capturing the episodes. Another explanation is that a longer window length can improve generalization across a variety of restraint forms. Infants can be restrained in various ways—such as in stationary seats, jumpers, swings, or caregivers’ arms, either still or while being carried around. Different restraint forms are presented by different motion features. By encompassing a broader range of movement patterns, a longer window can provide more informative and generalizable features for the model to account for variability within the restrained class. Given a restrained bout usually lasts minutes rather than seconds, a 30-s window length presents a good balance.

Our analysis of window length informs future modeling studies to consider temporal resolution as a behavior-informed decision. In infants’ everyday activities, behaviors unfold across various timescales—from second-by-second movement (e.g., locomotion) to longer periods of states (e.g., on the ground vs. lifted). Our optimal window length for restraint detection turns out to be longer than the window lengths in other models for body position classification (Airaksinen et al., 2020; Franchak et al., 2021, 2024a, 2024b; Yao et al., 2019), corresponding to the fact that restrained bouts last longer than position bouts. We recommend selecting window lengths that align with the typical duration and transition of the targeted behavior in everyday life.

**Fig. 6:**
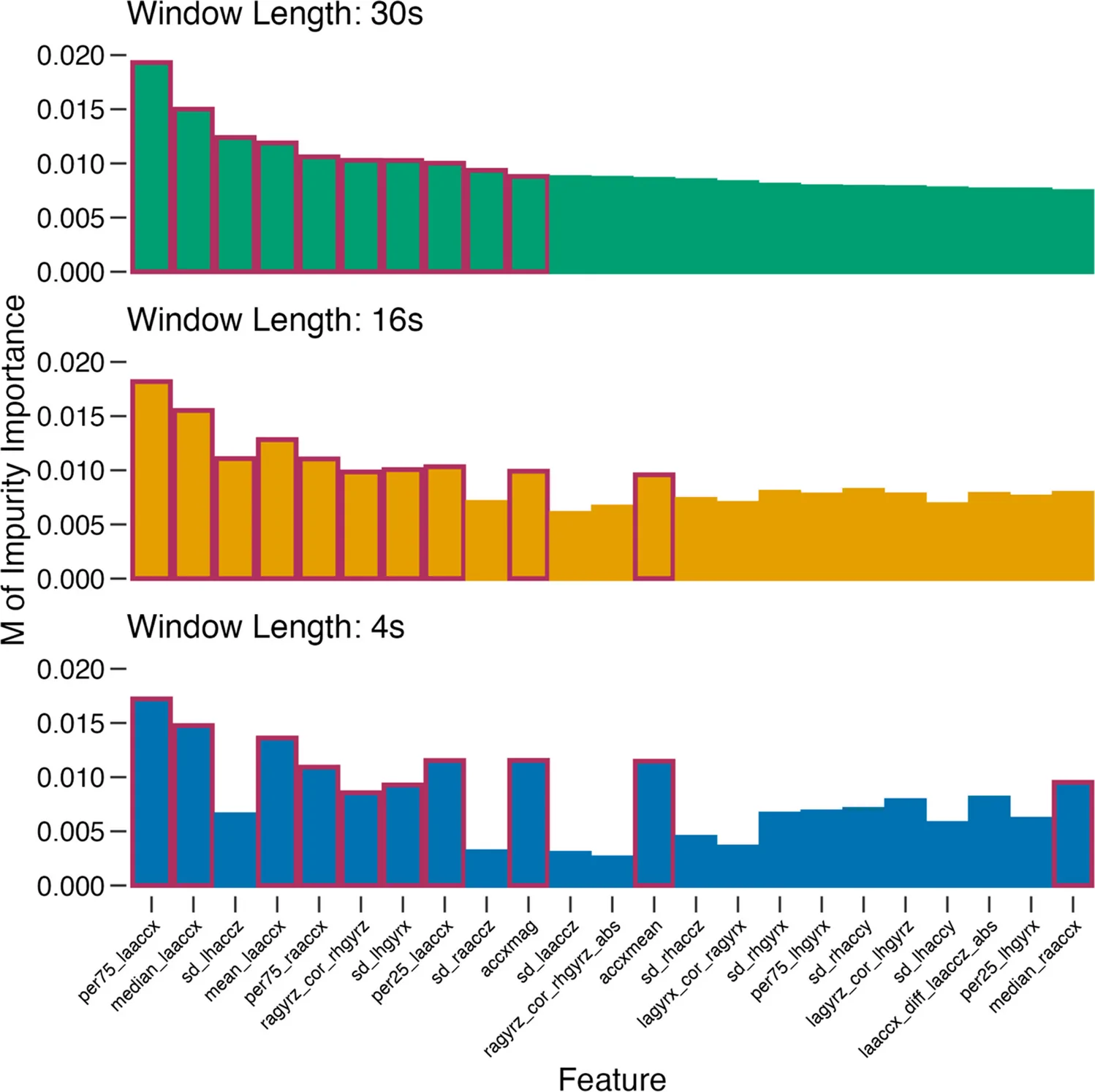
Feature importance by window length. *Note.* Feature plotted include the top ten features for each of the three window lengths out of the total 436 features. Each *bar* represents one feature. Feature names use the following abbreviations to indicate the location, sensor, and axis: “la” stands for left ankle, “ra” stands for right ankle, “lh” stands for left hip, and “rh” stands for right hip. “acc” stands for “accelerometer” and “gyr” stands for “gyroscope”. “*x*” “*y*” and “*z*” are axes in the accelerometer or gyroscope. The features are ordered along the* x*-axis based on their importance in the 30-s window model. The *bars with red* outline are the top ten features for each window length

We also inspected the top-ranked features in the model, which suggests that restraint classification relied on the signals from all four sensors, from both accelerometers and gyroscopes, and from various kinds of motion feature calculations (e.g., mean, median, SD, 75th percentile, correlation between sensors, magnitude). The top ten features for each window length are reported in Appendix A, Fig. 6. However, the feature ranking should be interpreted cautiously. This does not mean other features were not important, since a large number of total features (436) is likely to contain strong correlations between similar features. In addition, it should not be interpreted as evidence that the top-ranked features alone would be sufficient for model training. What the feature ranking does suggest is to use multiple sensors, use both sensor modalities, and incorporate different features, corroborating with previous work (Airaksinen et al., 2025; Franchak et al., 2024a, 2024b).

### Advances in methodology

The key contribution of this work is introducing a novel approach for measuring infant restraint in the home environment using wearable sensors. Wearable sensors offer several advantages over previous methods when quantifying day-long movement in naturalistic settings. Compared with survey methods, wearable sensors provide objective, continuous measurements with fine-grained temporal resolution. Compared with video recording and human coding methods, wearable sensors are less obtrusive and enable substantially longer recordings. With the machine learning model established, researchers can extend their investigation timescales to a full day or even multiple days at low cost. Although the current machine learning model achieves slightly lower accuracy (89%) than human annotation (99%), the trade-off between accuracy and human labor favors the sensor approach—especially when the target behavior fluctuates over longer timescales beyond the practical limits of human coding. Restraint is such a behavior that does not recur repeatedly every hour but follows the rhythm of daily routines and activities. Therefore, a wearable sensor system capable of generating restraint profiles across full days offers greater scalability for measuring infants’ everyday experiences.

de Barbaro et al. (2026) pointed out that machine learning models are constrained by their training data. Thus, the strong performance on the current dataset does not guarantee a comparable performance on new datasets. Applying the model to new populations or new settings (e.g., different inertial sensor brands) will require coding ground-truth data to check the model’s accuracy. In addition, the model may need to be re-tuned when applied to new restrained forms it has not encountered before. For example, some cultures involve distinctive forms of restraint, such as the Tajik “gahvora” (Karasik et al., 2018), Tseltal slings that may either fully wrap the infant or leave the head and torso free (Casey et al., 2026), and the use of sandbags or heavy blankets in other contexts (Adolph et al., 2010; Super & Harkness, 1986). These forms of restraint may produce hip and ankle movement patterns that were not represented in the current dataset, and whether the current model can detect them remains uncertain.

Moving beyond restraint, researchers should consider using the sensor system to capture a broader repertoire of infant behaviors—such as locomotion, steps, and falls. If proven feasible, the new models will substantially reduce the human labor required to code those behaviors. Furthermore, other than expanding the range of behaviors, future work should also broaden the scenarios in which wearable sensors are applied. To date, most wearable sensor applications have been limited to laboratory or in-home environments. Infants’ outdoor experiences remain largely underexplored, whereas outdoor environments provide infants with rich and unique opportunities for learning and exploration (Fjørtoft & Sageie, 2000; Herrington & Studtmann, 1998; Schneider, 2025). How much time do infants spend restrained in strollers, car seats, or in carriers worn by caregivers? Which positions are they frequently in when restrained out of the house? Validating sensor-based models in outdoor scenarios can open new avenues for investigation.

### Advances in understanding full-day real-time restraint

Methodological advances can deepen understanding of infants’ full-day, real-time restrained experiences. When and how to restrain infants is decided and imposed by caregivers, which in turn, shapes infants’ real-time experiences—such as when and how often infants have the opportunities for movement, which portions of the environment they can perceive, and what objects and people they can interact with. Thus, full-day restraint data can be used to answer two complementary questions: what factors shape caregivers’ moment-to-moment restraint decisions, and how do those decisions structure infants’ real-time sensorimotor experiences?

Prior work provided valuable insights into broad parental beliefs and attitudes of restraint. For example, parental concerns over floor play, beliefs about physical activity, and practices of co-play are found to be associated with restraint frequency and infants’ physical activity level (Hänisch et al., 2025; Hesketh et al., 2015; Hnatiuk et al., 2013; Karasik, 2025; Prioreschi et al., 2017). However, few studies have examined how each restraint decision is formed in real time–when caregivers choose to restrain or release an infant, what immediately precedes those decisions, and how these decisions vary across infants, caregivers, and contexts. For example, restraint decisions may vary with infant characteristics (e.g., motor skill, activity level, attachment style, or emotion expression), caregiver characteristics (e.g., beliefs, stress, habits), family daily routines, and broader socio-cultural factors, such as cultural norms (Karasik et al., 2015, 2018). By locating each individual bout of restraint, the current system enables researchers to examine restraint not only as a static summary metric, but also as a dynamic caregiving practice embedded in everyday life.

On the other hand, a full-day continuous profile of restraint can also help understand how infant real-time sensorimotor experiences are embedded in different restrained and unrestrained periods. An overall estimate of restrained time is not enough to examine the mechanisms between restraint practices and developmental outcomes. Take motor development as an example. Karasik et al. (2023) found that Tajik infants achieved motor milestones later than US infants, even though both groups share comparable amounts of overall restrained time outside of naps (Karasik et al., 2022). However, they also found that Tajik infants had more prolonged individual restrained bouts (Karasik et al., 2022), suggesting that how the overall restrained time is distributed across the day is important to investigate. Moreover, restraint timing (i.e., when infants are restrained) can be linked to other real-time experiences, such as speech input and social interaction. This raises questions about what learning opportunities are offered or lost during restrained periods, how those opportunities are distributed across a day, and what distribution is optimal for infant learning. Additionally, restraint timing can also be linked to infants’ self-initiated explorations. This approach can also answer questions such as how infants’ vocalizations, attention, locomotion, or object interactions differ across restrained and unrestrained periods, and whether opportunities lost during restraint can be compensated for in later unrestrained periods. In short, full-day quantification of infant restraint offers a foundation for linking caregiver practices to infants’ moment-to-moment sensorimotor experiences, thereby shaping infants’ developmental outcomes.

### Limitations and future directions

One limitation of the current study was its reliance on parent reports to exclude infants’ naps and times when the garment was removed. Caregivers may forget to log these periods or may record imprecise times, and we did not have an independent way to verify the accuracy of parent logs. Future work should incorporate objective approaches to improve the identification of these periods. For example, Bang et al. (2023) developed an algorithm to detect sleep periods from LENA (https://www.lena.org/) audio recordings. Although automated detection, like any machine learning predictions, is not perfect, using multiple sources of information—such as caregiver logs, audio-based sleep detection, and potentially, sensor-based movement detection—could allow for cross validation and more reliable estimates of infants’ awake wearing time.

Another limitation lies in the lack of contextual information that prevents us from drawing definite developmental conclusions. Restraint takes on various forms, and even the same form of restraint can bring distinct experiences for infants. Detecting *when* restraint happens does not grant knowing *what* experiences occur. For example, an infant placed in a highchair while their caregiver provides face-to-face feeding interaction would receive rich linguistic and social input. In contrast, an infant placed in the same device while the caregiver is occupied with household chores would experience a less stimulating, interactive environment. To map restraint onto broader developmental outcomes, future research should integrate complementary data streams that capture the concurrent situational context. Incorporating multimodal tracking—such as audio recordings and caregiver-infant proximity detection (Salo et al., 2022)—will provide the vital environmental backdrop needed to fully interpret restrained experiences.

## Conclusion

To conclude, the current study provided a powerful tool for quantifying infants’ everyday restrained experiences in the home using wearable sensors and machine learning modeling. The model achieved high accuracy and strong agreement with human annotations, as well as convergent validity by showing an age-related decrease in the overall restrained time. We explored the window length in the modeling procedure and demonstrated that a longer window enhanced the model’s balanced performance. Together, the current study advanced the method for measuring infant full-day, real-time restrained experiences, and opened avenues for research on how restraint imposed by caregivers can shape early development.

## Data Availability

Sensor data and video recordings are publicly shared on Databrary (Franchak, 2023). Compiled human annotation results are shared on OSF (https://osf.io/a698g/overview?view_only=cc1483debc864757a99a059bb4b055d1).

## Code availability

All processing scripts, including model training and validation analyses, are publicly shared on OSF as well (https://osf.io/a698g/overview?view_only=cc1483debc864757a99a059bb4b055d1).
